# Supplementary material for: Deep learning enabled smart mats as a scalable floor monitoring system
Source: Nat Commun. 2020 Sep 14;11:4609. doi: 10.1038/s41467-020-18471-z (PMC7490371; doi:10.1038/s41467-020-18471-z)
Supplement: Supplementary file 1 — Supplementary Information [file 41467_2020_18471_MOESM1_ESM.pdf]

Supplementary Information

Deep learning enabled smart mats as a scalable floor monitoring system

Shi et al.

Supplementary Figure 1. The gradual printing results using the same printing mask (20%).

Supplementary Figure 2. The operation mechanism with detailed charge transfer processes.

Supplementary Figure 3. Characteristics of the output performance of the individual DLES-mat on a 100 MΩ external load.

Supplementary Figure 4. The detailed output voltage waveforms on different external loads (100% DLES-mat).

Supplementary Figure 5. Electrode connection scheme investigation with six different DLES-mats.

Supplementary Figure 6. The output signals for the normal walking and walking-falling process.

Supplementary Figure 7. The energy harvesting performance of the DLES-mat array and individual DLES-mats.

Supplementary Figure 8. The capacitor charging performance.

Supplementary Figure 9. The output voltage patterns of 10 different users.

Supplementary Figure 10. The accuracy variation with respect to the training epoch.

Supplementary Figure 11. The generated voltage patterns with different passing statuses through the middle row of the DLES-mat array.

Supplementary Figure 12. The individual recognition with different passing statuses.

Supplementary Table 1. The parameters for constructing the CNN model.

Supplementary Note 1. Cost estimation of the fabricated DLES-mats.

Supplementary Figures

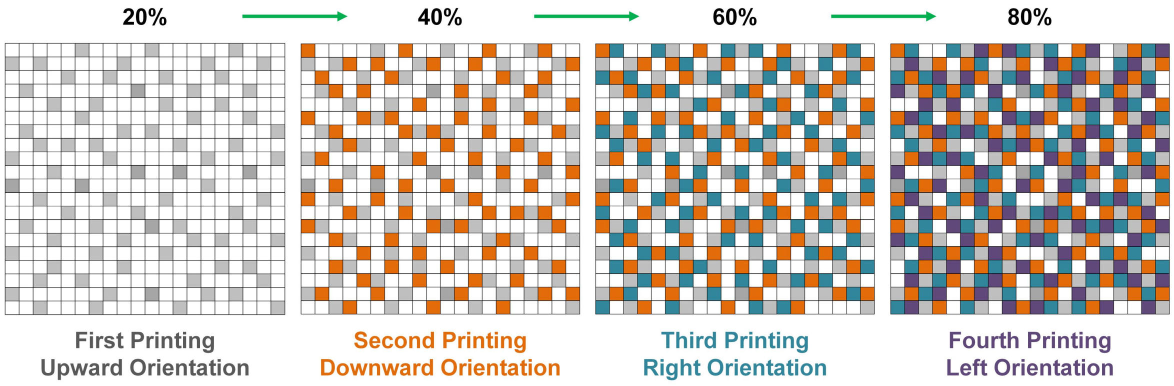

Supplementary Figure 1. The gradual printing results using the same printing mask (20%). The printing mask is oriented in different directions to achieve the 20%, 40%, 60%, and 80% deep learning enabled smart mats (DLES-mats). The different filled colors indicate the printing results from each printing.

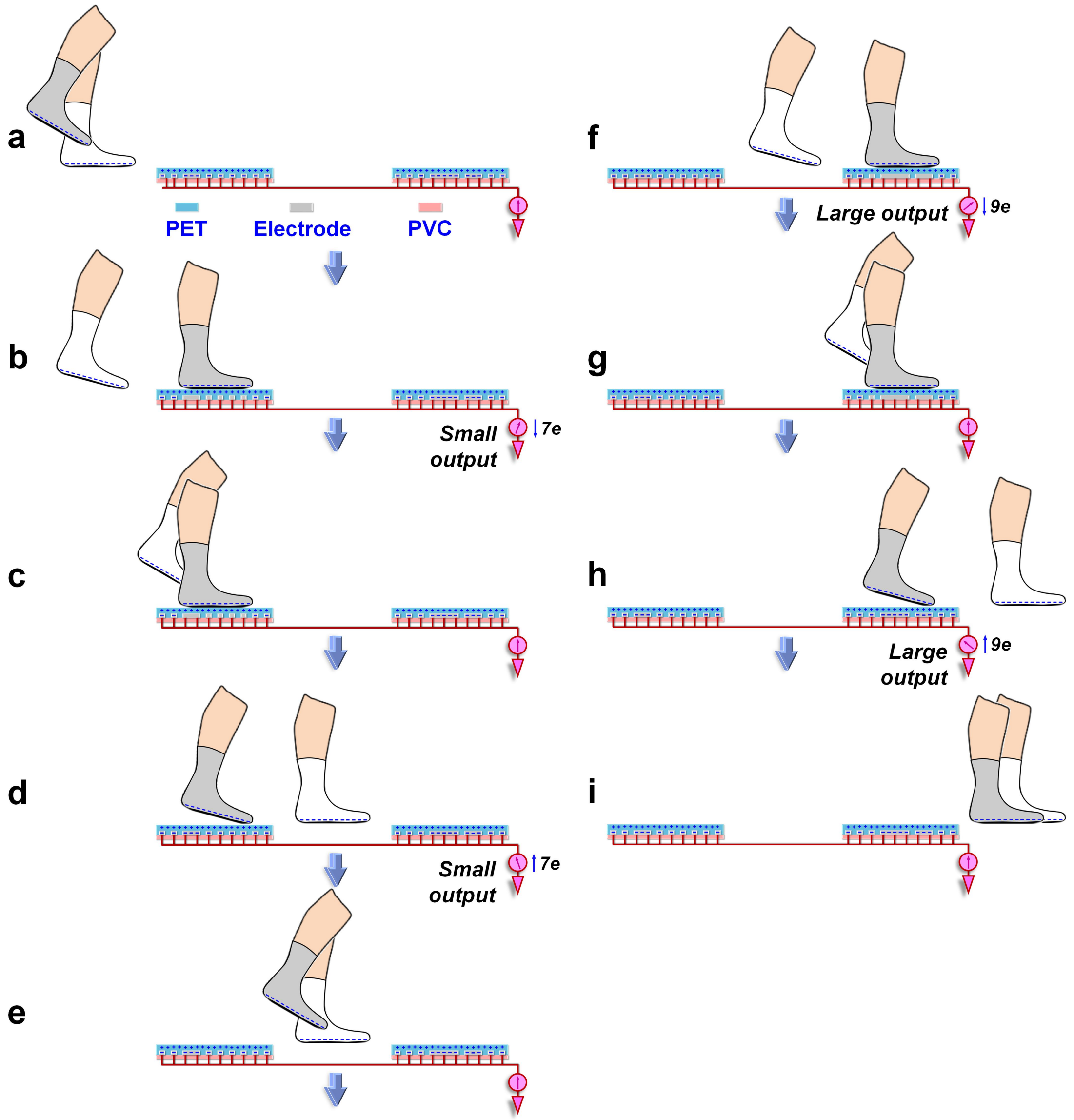

Supplementary Figure 2. The operation mechanism with detailed charge transfer processes. a-i The operation mechanism corresponding to different human walking phases when a person walks on and off the parallel-connected DLES-mats with a relatively smaller and a relatively larger electrode coverage rate, where the output signals with a relatively smaller and a relatively larger magnitude are generated, respectively.

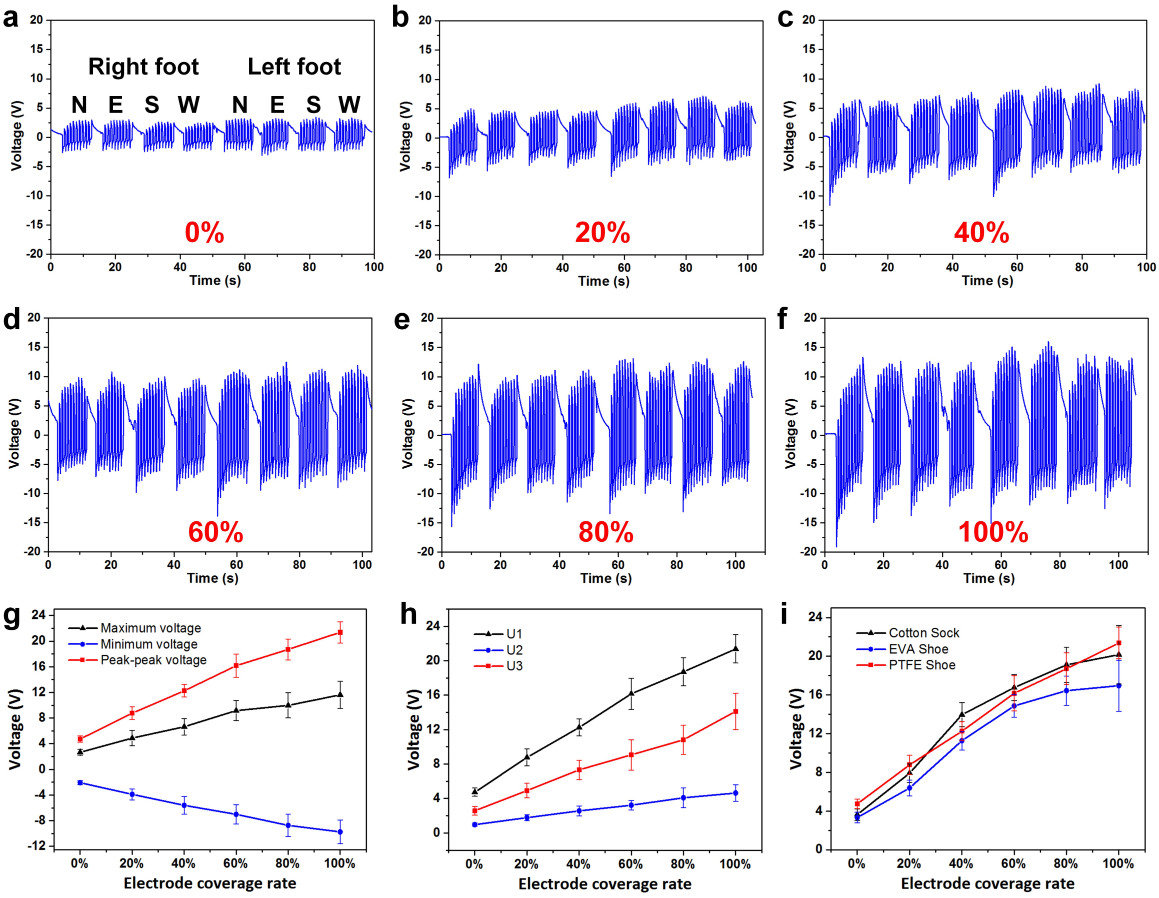

Supplementary Figure 3. Characteristics of the output performance of the individual DLES-mat on a 100 MΩ external load. a-f The generated voltages by repeated stepping motions with polytetrafluoroethylene (PTFE) shoes (four directions toward the north, east, south and west for both the right foot and the left foot) on DLES-mats with an electrode coverage rate of **a** 0%, **b** 20%, **c** 40%, **d** 60%, **e** 80%, and **f** 100%. **g** The corresponding maximum voltage, minimum voltage, and peak-to-peak voltage with respect to different electrode coverage rates. **h** The effect of different users wearing PTFE shoes on the output peak-to-peak voltage. **i** The effect of different contact materials worn by the same user on the peak-to-peak output voltage.

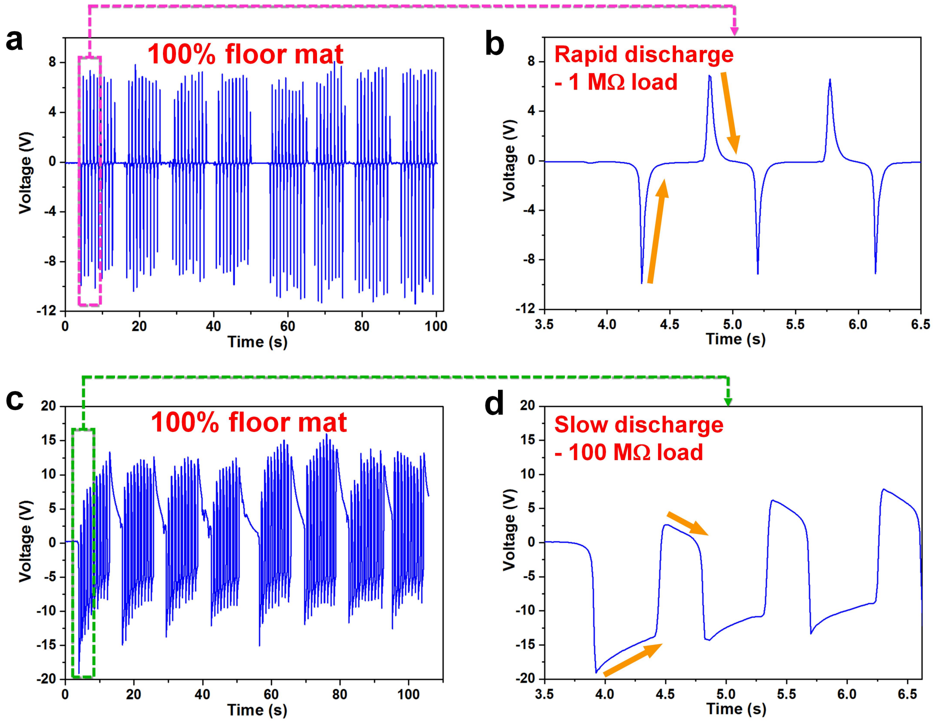

Supplementary Figure 4. The detailed output voltage waveforms on different external loads (100% DLES-mat). a-b Common and zoomed-in waveforms of the output voltage on a 1 MΩ load, showing the rapid discharge time due to the small resistance in the RC discharge process. **c-d** Common and zoomed-in voltage waveforms of the output voltage on a 100 MΩ load, showing much slower discharge time due to the large resistance in the RC discharge process.

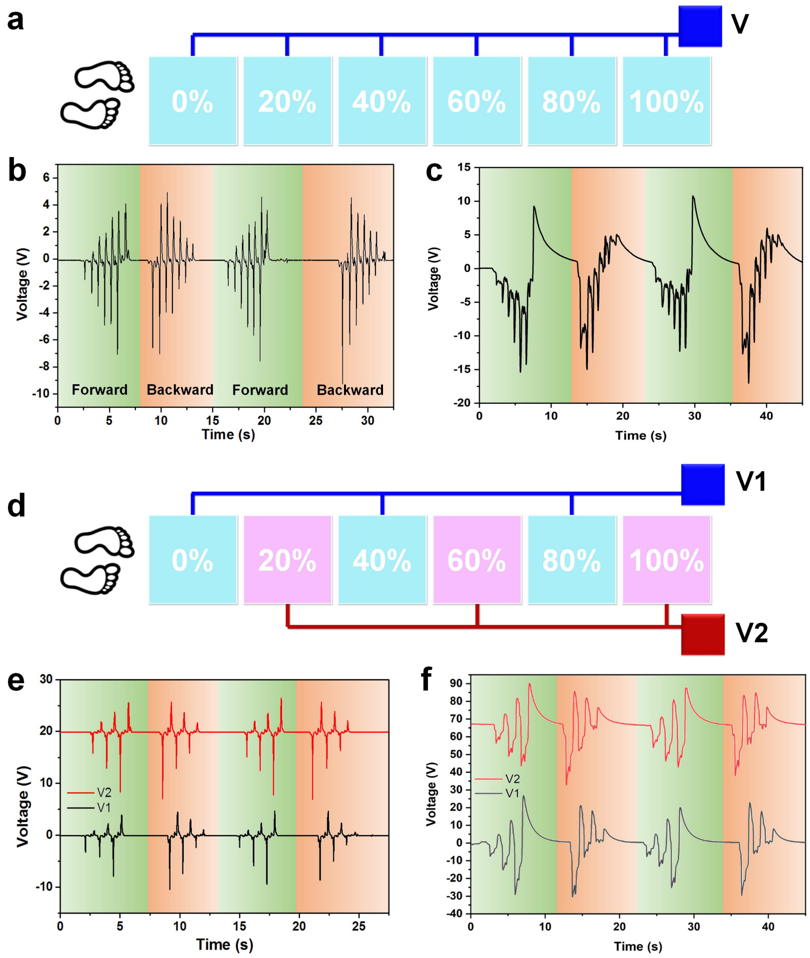

Supplementary Figure 5. Electrode connection scheme investigation with six different DLES-mats. a The schematic diagram of parallel connection of all the DLES-mats into one output electrode. **b-c** The generated output voltage on **b** a 1 MΩ external load and **c** a 100 MΩ external load with two rounds of forward-backward walking. **d** The schematic diagram of interval parallel connection of all the DLES-mats into two output electrodes to eliminate the adjacent interference. **e-f** The generated output voltage on **e** a 1 MΩ external load and **f** a 100 MΩ external load with two rounds of forward-backward walking.

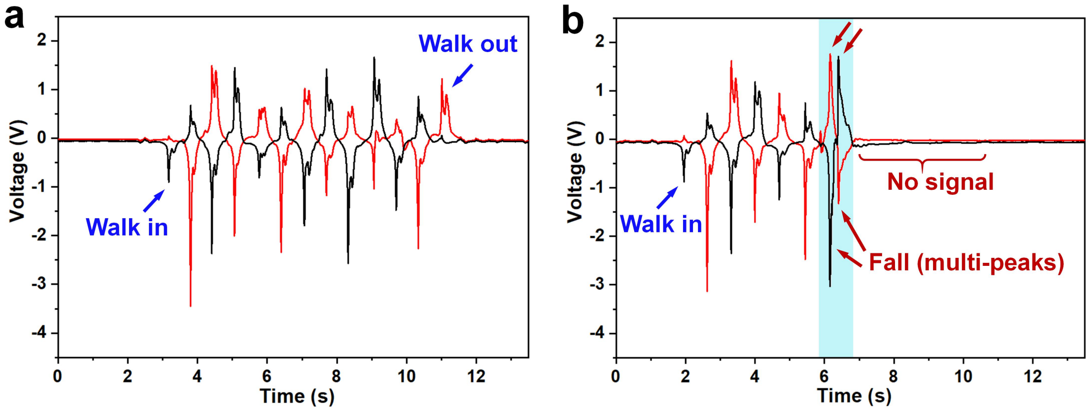

Supplementary Figure 6. The output signals for the normal walking and walking-falling process. The typical signal patterns for **a** the normal walking process and **b** the walking-falling process on the DLES-mat array. When falling, multiple output peaks are generated in a short period due to the falling induced rapid contacts with the DLES-mat array, and no outputs are generated after that.

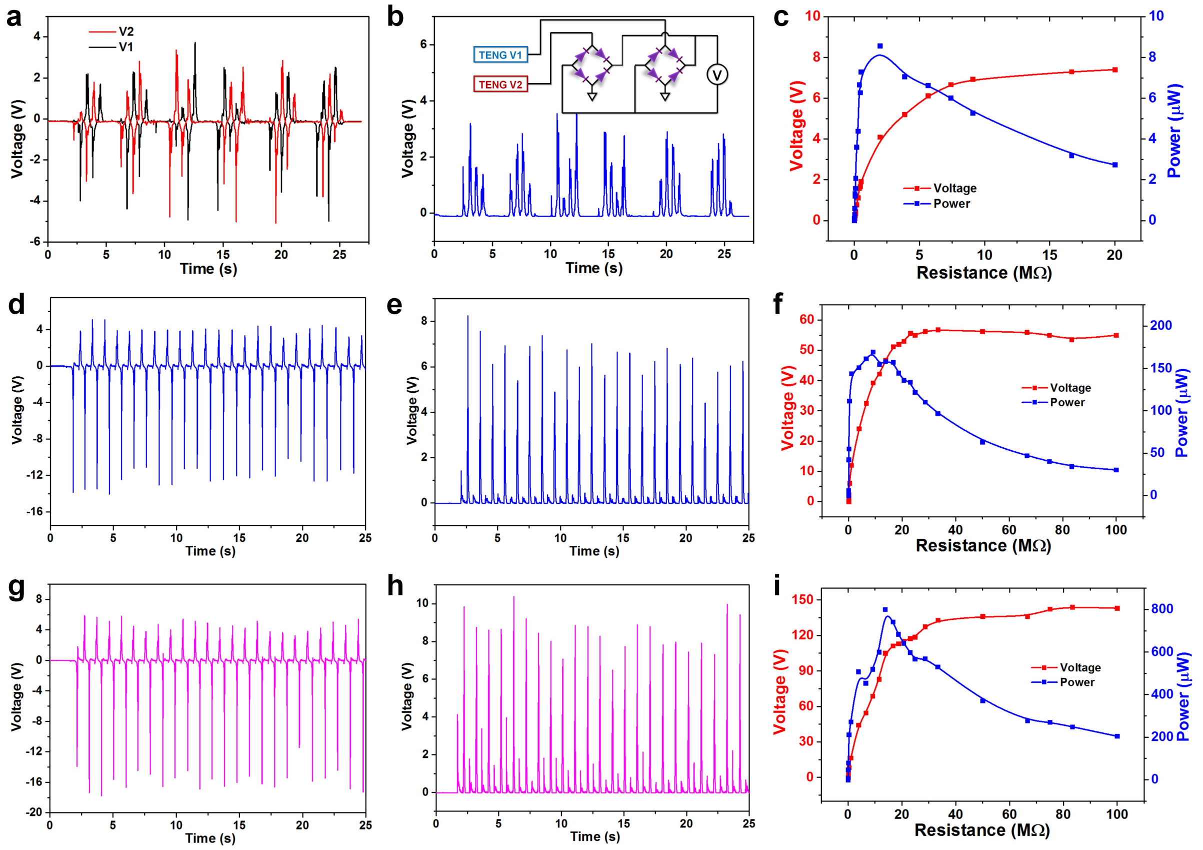

Supplementary Figure 7. The energy harvesting performance of the DLES-mat array and individual DLES-mats. a-b The generated output voltage of the 3 × 4 DLES-mat array on a 1 MΩ external load **a** before and **b** after rectification (with the inset showing the circuit connection diagram). **c** The output voltage and output power of the 3 × 4 DLES-mat array with respect to different external resistance. **d-e** The generated output voltage of a 40 cm × 40 cm DLES-mat on a 1 MΩ external load **d** before and **e** after rectification. **f** The output voltage and output power of the 40 cm × 40 cm DLES-mat with respect to different external resistance. **g-h** The generated output voltage of a 30 cm × 12 cm DLES-mat on a 1 MΩ external load **g** before and **h** after rectification. **i** The output voltage and output power of the 30 cm × 12 cm DLES-mat with respect to different external resistance. Higher output voltage and power can be achieved with reduced mat size, while the matched load resistance to obtain maximum power also increases.

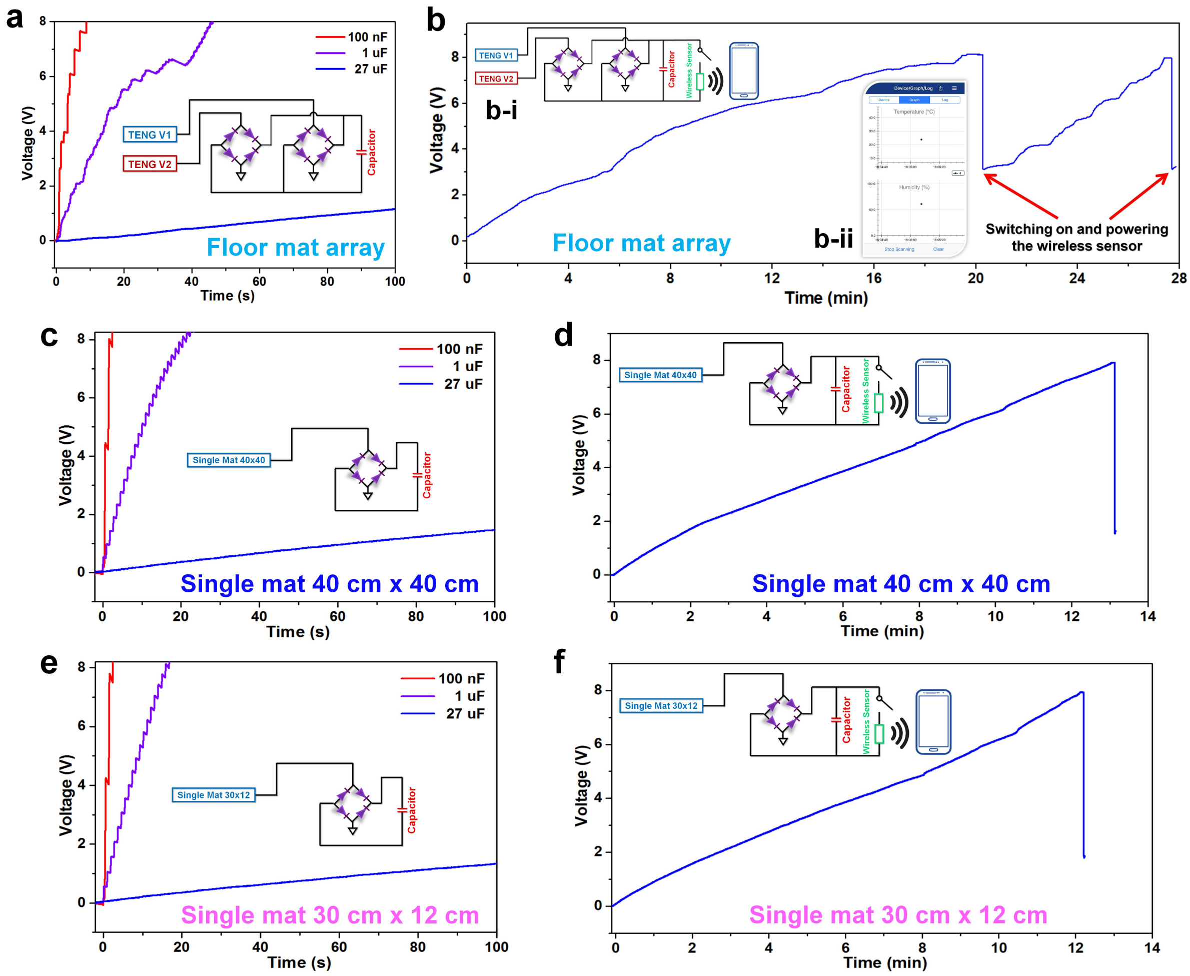

Supplementary Figure 8. The capacitor charging performance. a The capacitor charging curves of the 3 × 4 DLES-mat array with repeated stepping. The inset shows the connection diagram of the charging circuit. **b** Using the stored energy in the 27 μF capacitor (charged up to 8 V) to support the operation of a temperature/humidity sensor module with wireless data transmission. Inset (i) shows the constructed circuit diagram and inset (ii) shows the received sensing data on the smartphone end. **c** The capacitor charging curves of the 40 cm × 40 cm DLES-mat with the same repeated stepping. **d** Using the stored energy in the 27 μF capacitor to support the operation of the temperature/humidity sensor. **e** The capacitor charging curves of the 30 cm × 12 cm DLES-mat with the same repeated stepping. **f** Using the stored energy in the 27 μF capacitor to drive the temperature/humidity sensor.

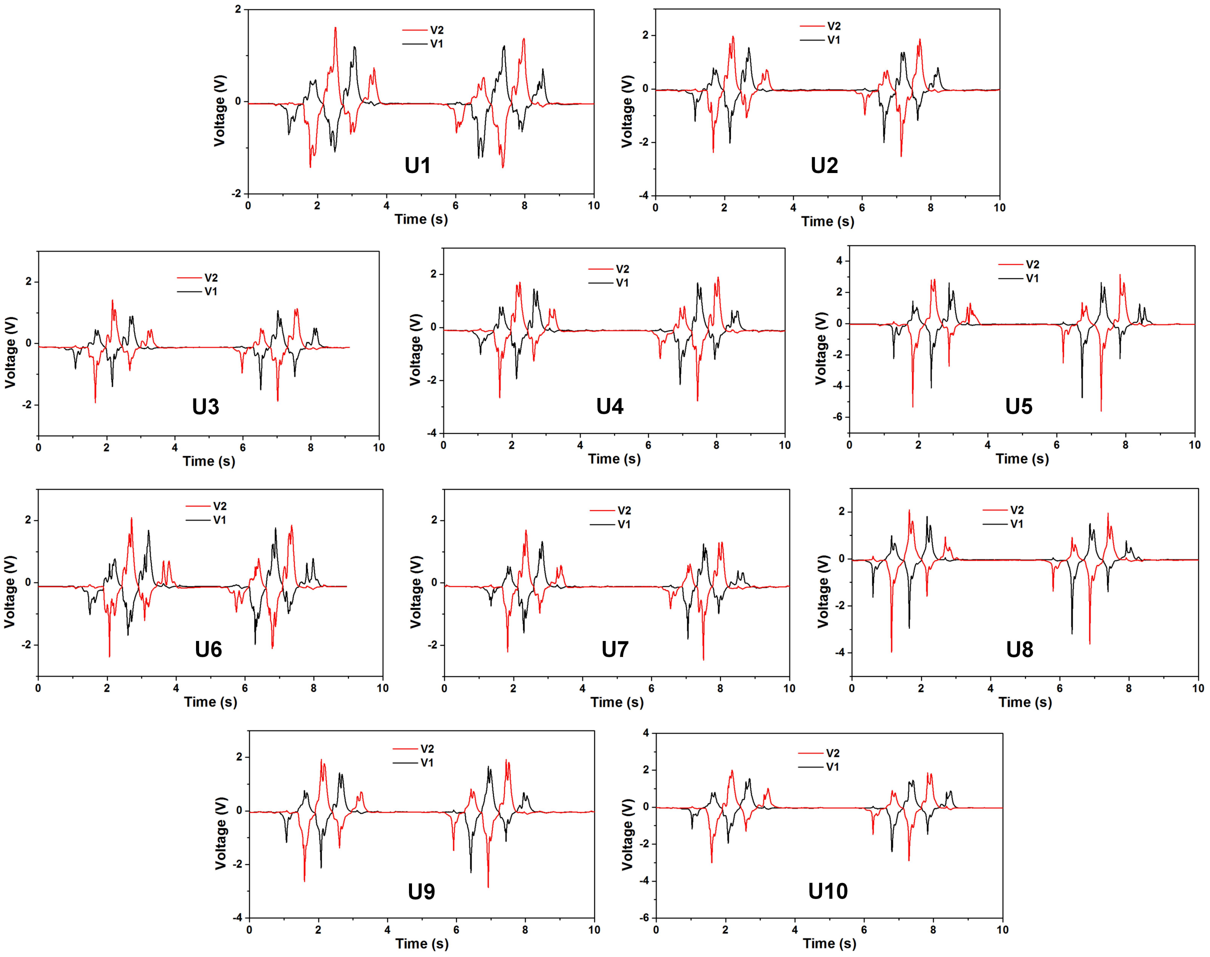

Supplementary Figure 9. The output voltage patterns of 10 different users. The generated voltage patterns of 10 different users walking through the middle row of the 3 × 4 DLES-mat array (E1 20% → E2 80% → E1 60% → E2 20%) in a forward-backward manner.

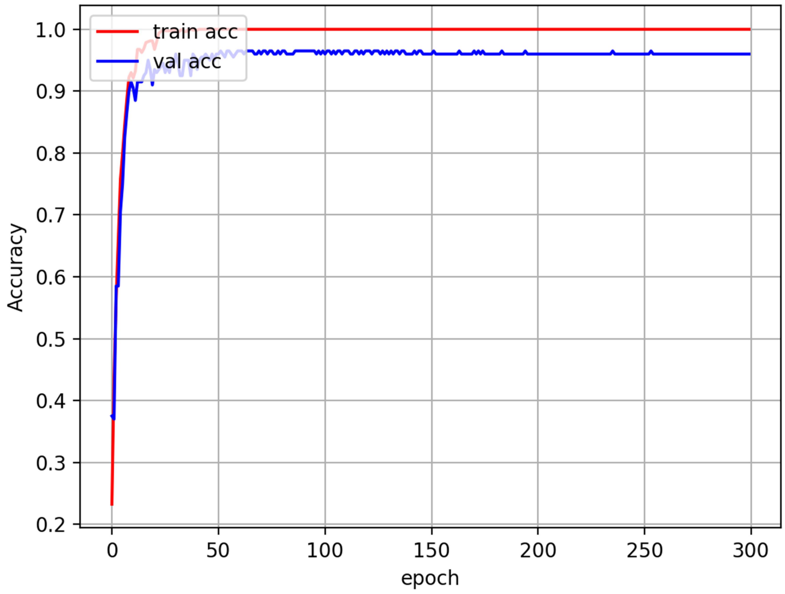

Supplementary Figure 10. The accuracy variation with respect to the training epoch. After the training process with 50 training epochs, the maximum accuracy can be achieved, and the convolutional neural network (CNN) model is able to generalize enough to avoid overfitting.

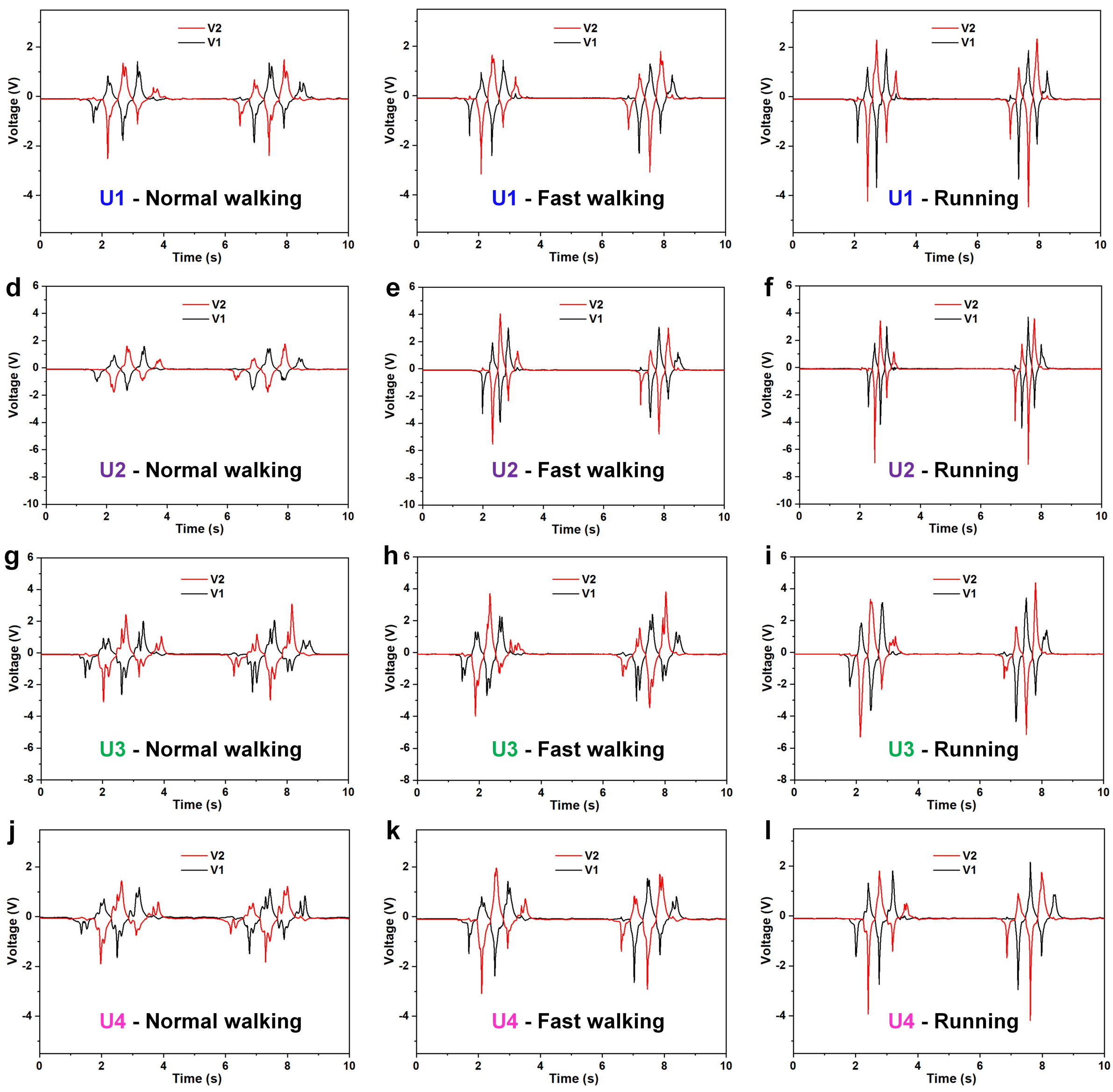

Supplementary Figure 11. The generated voltage patterns with different passing statuses through the middle row of the DLES-mat array. a-c The output voltage patterns from User 1 (U1). **d-f** The output voltage patterns from User 2 (U2). **g-i** The output voltage patterns from User 3 (U3). **j-l** The output voltage patterns from User 4 (U4).

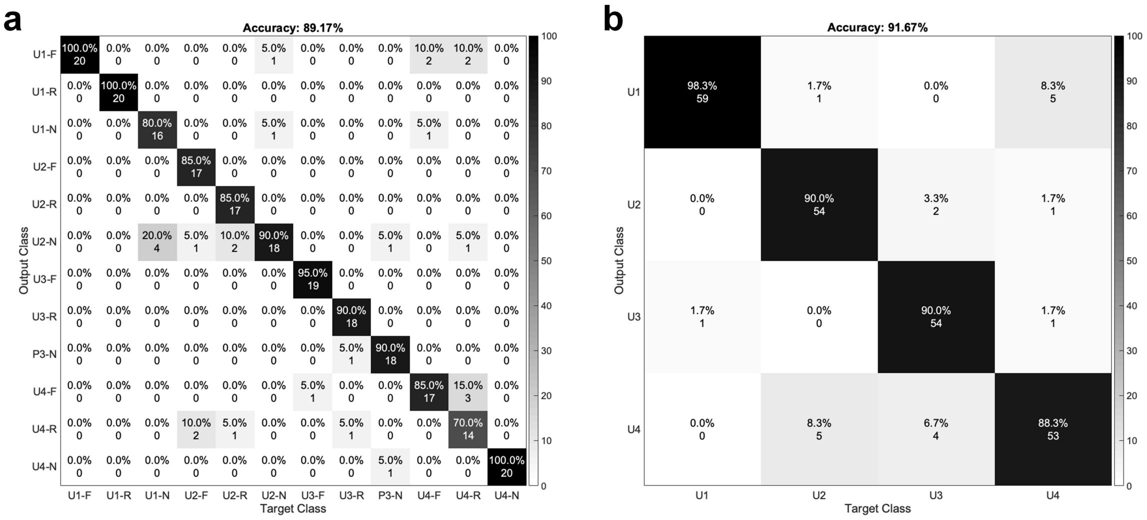

Supplementary Figure 12. The individual recognition with different passing statuses. The confusion matrix for individual recognition in different statuses with **a** known passing status (N, normal walking; F, fast walking; R, running), and **b** unknown passing status.

Supplementary Tables

Supplementary Table 1. The parameters for constructing the CNN model

No	Layer Type	No. of Filters	Kernel/ Pool Size	Stride	Output Size	Padding
1	Convolution 1	32	50	1	(None, 1600, 32)	Same
2	Max Pooling 1		20	2	(None, 800, 32)	Same
3	Convolution 2	64	10	1	(None, 800, 64)	Same
4	Max Pooling 2		20	2	(None, 400, 64)	Same
5	Convolution 3	128	10	1	(None, 400, 128)	Same
6	Max Pooling 3		20	2	(None, 200, 128)	Same
7	Convolution 4	256	20	1	(None, 200,256)	Same
8	Max Pooling 4		10	2	(None, 100,256)	Same
9	Flatten				(None, 25600)	Same
10	Dense (input)				(None, 3200)	
11	Dense (output)				(None, 10)	

Supplementary Notes

Supplementary Note 1. Cost estimation of the fabricated DLES-mats mats

According to our estimation on the consumed materials (including Ag, PET, PVC, etc.) and the fabrication expenditure which can be significantly reduced in mass production, the actual cost for one set of the DLES-mats (including 0%, 20%, 40%, 60%, 80%, and 100% mats) is about US$28.7. Hence the average cost for one DLES-mat with a large sensing area of 40 cm × 40 cm is around US$4.8.
