# Supplementary material for: Deep learning enabled smart mats as a scalable floor monitoring system
Source: Nat Commun. 2020 Sep 14;11:4609. doi: 10.1038/s41467-020-18471-z (PMC7490371; doi:10.1038/s41467-020-18471-z)
Supplement: Supplementary file 3 — Description of Additional Supplementary Files [file 41467_2020_18471_MOESM3_ESM.docx]

**Description of Additional Supplementary Files**

**File Name: Supplementary Movie 1**

**Description:** The smart building demonstration based on the smart floor monitoring system for position sensing and individual recognition of three different users.
